# The Social Construction of Infertility Among Iranian Infertile Women: A Qualitative Study

**Published:** 2019

**Authors:** Syedeh Batool Hasanpoor-Azghady, Masoumeh Simbar, Abou Ali Vedadhir, Seyed Ali Azin, Leila Amiri-Farahani

**Affiliations:** 1- Department of Reproductive Health and Midwifery, Nursing Care Research Center (NCRC), School of Nursing and Midwifery, Iran University of Medical Science, Tehran, Iran; 2- Midwifery and Reproductive Health Research Center (MRHRC), Department of Midwifery and Reproductive Health, School of Nursing and Midwifery, Shahid Beheshti University of Medical Sciences, Tehran, Iran, Department of Midwifery and Reproductive Health Research Center, Shahid Beheshti University of Medical Sciences, Tehran, Iran; 3- Department of Anthropology, Faculty of Social Sciences, University of Tehran, Tehran, Iran; 4- Reproductive Biotechnology Research Center, Avicenna Research Institute, ACECR, Tehran, Iran

**Keywords:** Grounded theory, Infertile women, Infertility, Social construction

## Abstract

**Background:**

Infertility is considered an important phenomenon in couples’ life. Infertility and its treatment process influence all aspects of the individual’s life. This study aimed to explain the psycho-social process of social construction of infertility among Iranian infertile women.

**Methods:**

This was a qualitative study using a grounded theory approach. The study setting was the Vali-e-Asr Fertility Health Research Center and Avicenna Fertility clinic in Tehran. The sampling started purposefully and it was continued theoretically. The data collection was performed by using 36 semi-structured interviews, observation and field notes with 27 women who suffered from primary and secondary infertility having no living child. The method suggested by Strauss and Corbin was used for data analysis.

**Results:**

Results indicate that “Concerns over life instability” and “being judged by others” were the participants’ most important preoccupation. Attempts to stabilize life and get rid of being judged by others were key aspects of the social construction of infertility and the main strategies for resolving their preoccupation. This core concept explained the basic psychological-social process of infertility in relation to axial codes.

**Conclusion:**

The results of the study show that various interactive factors affect the social construction of infertility among infertile women who focus on the central concept of attempts to stabilize life and get rid of being judged by others. Therefore, in order to achieve this goal, infertile women should be empowered by effective coping strategies.

## Introduction

Infertility is considered a debilitating problem with negative effects on public health (1). Advancements in infertility treatment interventions in the 20th century are described as a double-edged sword that may create psychological, social, ethical, financial and legal problems (2).

It has been reported that about 10% of the world populations are infertile (3). According to the findings of an Iranian study, the prevalence of infertility was 20.2%, which was higher than the global prevalence rate (4).

Infertility is associated with a wide range of social, psychological, physical and financial problems for couples (5, 6). The problem of infertility in today’s world has become a social concern that leads to a psychological imbalance between couples and sometimes interrupts their relationship (7). WHO has identified infertility as a major problem in reproductive health (8), while there is a general agreement that the women’s role and status should not be defined merely based on their fertility capacity; in many societies, the feeling of femininity is understood through being a mother, which is often the only tool for women to advance their status within both the family and society (9). In some societies, infertility is perceived as the gender-related suffering and mainly a women-related problem (10). Infertile women with successive failures in childbearing experience higher levels of stress and anxiety (11). It is believed that infertility influences women rather than men (12, 13).

In the Iranian context, a special socio-cultural and religious significance is given to childbearing. The Iranian culture considers children as divine blessings and childlessness as unpleasant. This significance is also reflected in some of the policy documents including the Iranian Family Protection Act which stresses the importance of child-bearing because infertility is considered a cause of divorce (14). In the Iranian culture, patriarchal beliefs for survival and generation continuity, lack of social and economic support for most women, low chance of remarriage for an infertile woman, and condemnation of solitary life double concerns arising from infertility among women (15). An outstanding difference is present between couples’ infertility experiences in developed and developing countries. In developed countries, infertility is considered voluntary; women have the right not to have children and are assumed as “childfree” (12).

Infertility is often understood as a phenomenon with medical, ethical or mental aspects. Therefore, little attention is given to the sociocultural context of infertility (16). It is believed that infertility experiences are influenced by sociocultural factors, the definition given by the community and how to treat infertile individuals and couples. Therefore, empowering women should be based upon a deep understanding of the infertility within the context of the society, in order to guide public policy and determine the direction for designing programs in the social sphere (2, 12).

Therefore, this study aimed at studying the social construction of infertility among infertile women seeking treatment in the Iranian cultural-social context.

## Methods

The present study aimed to explore main components influencing infertile women’s behaviors and understand cultural-social conditions affecting them using a grounded theory approach.

Participants were infertile women with primary and secondary infertility. The study setting was the Vali-e-Asr Fertility Health Research Center and Avicenna Fertility clinic in Tehran. The data collection and analysis lasted from August 2017 to March 2018. Infertile couples were referred to these centers for treatment from different parts of the country.

Inclusion criteria were suffering from primary or secondary infertility with only female cause, having no living child from secondary infertility, having no adopted children, lack of chronic and mental diseases and willingness to take part in this study. Sampling was first initiated based on the above-mentioned characteristics as a purposeful sampling method, and then by analyzing the data for the purpose of clarifying and developing emerging categories and formed questions. Sampling was directed with infertile women who have provided richer more in-depth experience to theoretical questions related to emerging categories. The choice of each participant in the theoretical sampling depends on the previous participants and their data extracted, and the relationship between data gathered from different participants. As the process of data collection and analysis proceeded and the categories and subcategories formed, theoretical sampling was directed based on them. The process of theoretical sampling continued until a new concept wasn’t created. Theoretical sampling led to a maximum variation in terms of age, duration of the marriage, duration of infertility, type of infertility, duration of treatment, education level, job and residence (Table 1).

**Table 1.** The demographic characteristics of the participants

**Variable**	**Mean**	**Number (%)**	**Range**
**Age (year)**	31±6.45		21–48
**Duration of marriage (year)**	7±4.53		2–22
**Duration of infertility (year)**	5±3.85		1–14
**Duration of treatment (year)**	3±3.32		1–14
**Education level**			
	Illiterate	1(3.7%)	
	Diploma and lower	13(48.1%)	
	Academic	13(48.1%)	
**Occupation**			
	Housewife	15(55.6%)	
	Employee	11(40.7%)	
	Retired	1(3.7%)	
**Type of infertility**			
	Primary	19(70.4%)	
	Secondary	8(29.6%)	

For data collection, semi-structured interviews and observations were held and field notes were taken. The interviews were started using the following question: “How did you find out that you have problems with fertility?” Next, the interviews were continued using exploratory questions.

Field notes were taken to confirm and strengthen the findings from the interviews. The categories’ theoretical saturation was reached with 25 participants. However, interviews were conducted with two more participants to ensure theoretical saturation. In total, 36 interviews were performed with 27 participants. Nine participants were interviewed twice to remove misperceptions during the coding process and ambiguous issues.

All participants were willing to be interviewed at the fertility centers. The participants were explained about the aim and method of the study, confidentiality and anonymity throughout data collection and analysis. If they were willing to participate in the study, they signed the written informed consent form. Each interview lasted for about 45–70 *min* with a mean of 60 *min*. The complementary interviews lasted for 15–20 *min* and were conducted on the phone. The tape-recorded interviews were transcribed verbatim and read several times to achieve a general understanding of the study phenomenon. MAXQDA10 software was used for data management.

The method suggested by Strauss and Corbin (17) was used for data analysis. This is a systematic, yet continuous process of comparing data. A three-stage process of coding was used including open coding, axial coding, and selective coding. For selective coding, the whole process and central variable was described, which were the refined outcome of the primary codes. Using the model’s paradigm, categories were connected to the main category and the theory was developed.

To ensure rigor of the findings, variations in research participants, long-term involvement with the study, the phenomenon and research environment, various methods of data collection, coding-recoding method to ensure stability in data, simultaneous data collection and analysis were considered.

Moreover, the interviews and primary analysis were given to some infertile women and external reviewers to ensure that real aspects of phenomenon were presented in this study. Also, to examine transferability, the data and the story were given to four infertile women who did not participate in the study; their common feeling and suffering for the story were quite obvious.

### Ethical consideration:

This research project supported financially and was approved by the Ethics Committee affiliated with Iran University of Medical Sciences (Code: IR.IUMS.REC 95-04-28-3028).

## Results

Data analysis resulted in the development of 2156 initial codes. After excluding overlapped ones, 55 subcategories, 32 categories and 12 axial codes remained (Table 2).

**Table 2.** Summary of categories and subcategories

**Categories**	**Subcategories**
Couple’s interactions	The husband’s reactions and performance to infertility Hiding reproductive issues from one’s husband The effect of infertility on the verbal and emotional relationships of couples Sexual dissatisfaction due to infertility problems and treatment
Family and society’s judgment and performance	Family’s reaction and manner of dealing with infertility The society’s judgment and performance
Control of the treatment process over the life cycle	The tensions and stresses of continuing the treatment process The economic tensions of treatment
Personal beliefs and motivations for bearing a child	The individual’s beliefs and attitude towards infertility The individual’s motivation for childbearing The infertile woman’s attitude toward an adopted child
The psychological and social consequences of infertility	The psychological consequences of infertility The social consequences of infertility
The existence of social supporters against its absence	Social supporters The absence of supportive sources
The characteristics of an infertile woman	Individual biography and infertility characteristics Economic-social factors
Attempts to stabilize life and get rid of being judged by others (Core category)	Conducting treatment measures Conducting traditional treatments Spiritual and religious strategies Avoidance strategies Emotional- affective compatibility strategies Problem-solving strategies Hiding infertility from others
Hope for treatment interventions against its damages	Hope for treatment interventions The psychological consequences of the treatment process
The couple’s closer relationship against the threat to life instability	The couple’s closer relationship The likelihood of the disintegration of marital life
Relieving or adapting to psychological stress against its increase	Relieving or adapting to psychological stress Increased psychological stress
Spiritual growth against its challenges	Spiritual growth Challenging the individual’s spiritual beliefs

“Concerns over life instability” and “being judged by others’ were the participants” most important preoccupation. Attempts to stabilize life and get rid of being judged by others were key aspects of the social construction of infertility and main strategies for resolving their preoccupation. This core concept explained the basic psychological-social process of infertility in relation to axial codes. Couple’s interactions, family and society’s judgment performance, and the effect and control of treatment process over the life cycle were causal conditions of concerns, which initiated the process of stabilizing life and getting rid of being judged by others. Personal beliefs and motivations for childbearing and the psychosocial consequences of infertility (as the context) created conditions under which the participants went through this process. The characteristics of infertile women and the existence of social supporters against its absence were considered to be the intervening conditions facilitated or limited this process. Hope for treatment interventions against its damages, couples’ closer relationships against the threat to life instabilities, relieving or adapting to psychological stress against its increase and spiritual growth against spiritual challenges were the consequences of the process of attempting for life stabilization and getting rid of being judged by others.

### Couple’s interactions:

This category included four subcategories as follow:

1. ***The husband’s reactions and performance to infertility:*** Most participants believed that their husbands were supportive sources in different stages of the treatment process and consequences arising during the treatment process. However, some women were stigmatized by their husbands.
  Despite having financial abilities, the husbands of two participants were unwilling to pay treatment costs.
  “My husband often says that I am infertile and I am the source of the problem”. (23 years old, 1 year infertility).
  The prevalence of physical violence was low. However, some reported that they were physically tortured.
2. ***Hiding reproductive issues from one’s husband:*** Some participants who were deprived of their husbands’ supports attempted to hide the causes of infertility, treatment process or issues related to others’ childbearing from their husbands.
  “I suffer from the lazy ovary, but I have not told my husband yet. If he [husband] finds a very simple problem, he says that it is my fault”. (28 years old, 2 years infertility).
3. ***The effect of infertility on the dialogic and emotional relationships of couples:*** Reduced dialogic and emotional relationships were reported by both participants enjoying their husbands’ support and those who assumed their husband’s behavior as a main source of stress. It was emphasized when relatives attempted to interfere or infertility treatments failed.
  “When I was seeking pregnancy after the treatment, I got menstruated and he started arguing” (31 years old, 10 years infertility).
  The participants stated that their husbands were affected by mental issues when faced infertility and treatment processes, high costs of treatment, treatment failure and hearing about relatives’ pregnancy. In these conditions, the couple’s dialogic and emotional interactions were reduced or they started arguing.
4. ***Sexual dissatisfaction due to infertility problems and treatment:*** Some reported sexual dissatisfaction due to infertility problem and the treatment process.
  “Now, when I have a sexual relationship, I do not think of my husband’s feelings anymore. My husband is my life, but I think of something else”. (30 years old, 2 years infertility).

Family and society’s judgment and performance: The family, relatives, and close friends have affected the participants’ understandings and experiences. This category included two subcategories as follow:

1. ***Family’s reaction and manner of dealing with infertility:*** The participants asserted the role of emotional and financial support of their own families. However, the husband’s family had either a positive or negative supportive role. Emotional and financial support from the husband’s family was more frequently provided by families enjoying a higher educational level or stronger religious belief.
  “My husband’s family are very religious. They have strong beliefs. They are much better than me. They understand me”. (27 years old, 6 years infertility).
  Some participants who were stigmatized by their husband’s family had already lost their social status and were humiliated.
  “I was sitting with my husband’s grandmother when she said: ‘listen, dear, if you do not have any children next year at this time, I will burn you with this fire”. She said something that burned me inside” (22 years old, 6 years infertility).
2. ***The society’s judgment and performance:*** All participants, even those enjoyed husbands and family’s support sometimes were humiliated and blamed by relatives and close friends, that even their husband’s emotional support did not comfort them.
  “My mother-in-law holds a ceremony every year in “Muharram” [a religious month]. My sister-in-law and I serve guests. The guests pointed at me and started whispering” (30 years old, 7 years infertility).

Control of the treatment process over the life cycle: This category included two main subcategories as follow:

1. ***The tensions and stresses of continuing the treatment process:*** The stressful factors of the treatment process include the need for egg donation or surrogacy, unpredictability of the process and results of treatment, side effects of drugs and society’s negative attitudes towards treatment methods.
  “I do not tell anyone that I conducted IVF [*in vitro* fertilization]. They [relatives] stated that the baby was born from injecting an ampoule and that is not mine”. (29 years old, 3 years infertility).
  The treatment process interfered with participants and their husbands’ vocations and decisions for life plans.
2. ***The economic tensions of treatment:*** Treatment costs were high and most patients had no insurance. When their husbands could not finance treatment costs, it was difficult for them to ask for financial support from their families.
  “When my father paid treatment costs, my husband said: “I do not need his money. He has already paid too much. It is embarrassing”. (24 years old, 8 years infertility).

Personal beliefs and motivations for bearing a child: This category included three subcategories as follow:

1. ***The individual’s beliefs and attitude towards infertility:*** Most participants maintained that infertility was challenging in their lives, which was followed by complications such as questioning the meaning of life and facing ambiguity regarding the future of the couple’s life. They stated that the decision on the future of marital life was made by the person who had no fertility problem. However, some participants did not strongly believe in having any children in the marital life.
  “Fertility has always had its negative effects. It affects all aspects of my life [while crying after a long pause] “. (37 years old, 12 years infertility).
  Some participants stated that even a supportive husband could not understand them. Only an infertile counterpart was in the same conditions for the treatment process and could understand how they felt.
2. ***The individual’s motivation for childbearing:*** To most participants, childbearing created changes, varieties and happiness in life, helped escape from solitude and social pressure to life stability. Some wanted children to make their generation immortal and had heirs after their death. For some participants, children were assumed to be supportive sources for old age. For some others, children were considered to be complementary sources of identity.
  “The presence of a baby makes me feel stronger. It makes my life more stable as well”. (26 years old, 2 years infertility).
3. ***The infertile woman’s attitude toward an adopted child:*** The existence of blood-type relationships between children and parents was of great importance to some participants. Most participants preferred all kinds of medical interventions to adopt a child. Some participants maintained that the mother’s real love was expressed only for the biological child. Moreover, adopted children were likely to leave them in adulthood.
  “My love toward an adopted is not sincere and real; the child is not mine”. (31 years old, 2 years infertility).
  The short infertility duration and non-use of other medical methods were reasons for not thinking of adopting a child. Some others who had already thought of this issue were dubious about adopting a child.
  Some participants who searched about the duration and conditions of adopting a child stated that the legal conditions of adopting a child were too hard.
  Husbands influenced decisions over adopting a child. Some participants stated that their husbands considered this a kind of good deed and agreed to do so. However, many others were strongly against such an idea.
  “I do not dare to talk about adopting a child in front of my husband. He will stop talking to me”. (43 years old, 14 years infertility).
  It was stated that their husbands preferred remarriage rather than adopting a child. Reasons behind refusing to adopt a child included the adopted child’s psychological injuries by friends and relatives, the fear of disclosing the child’s adoption in the future by friends and relatives and people’s negative attitudes towards adoption.
  “When the child is not mine, everyone talks about me and the adopted child. I have twice as many problems as others have”. (36 years old, 4 years infertility).

The psychological and social consequences of infertility: This category includes two subcategories as follow:

1. ***The psychological consequences of infertility:*** The psychological consequences of infertility included mental involvement, turmoil, reduced patience, and tolerance, feeling of absurdity and aimlessness, lack of confidence, negative thoughts, distraction and blaming oneself, shock, fear, anxiety, negative feelings, sorrow, regret, and depression.
  “I was always involved with this issue that whether my problem would be resolved or not”. (30 years old, 2 years infertility).
  “It was too hard to believe that I have such a problem”. (31 years old, 2 years infertility).
  “Whenever I see a pregnant woman, I ask myself when that will happen to me? When can I put my hands on my belly too?” (26 years old, 2 years infertility).
2. ***The social consequences of infertility:*** The social consequences of infertility included stigma, social isolation, life instability, social exclusion, relative deprivation, and social alienation.
  While social isolation and life stability threats were social consequences, given the main question of the present study, they were included in other main categories and discussed elsewhere.
  Neglecting the participants and reduced interactions with relatives made them feel socially isolated.
  “My sister-in-law is now pregnant. I am really ignored. As if I do not exist at all”. (21 years old, 2 years infertility).
  Those participants who felt socially alienated were involved with a kind of confusion in guiding their behaviors and coordinating with social norms. This confusion was easily observed in how they treated others’ kids and pregnant women. It was also seen in how they behaved at some parties.
  “If I hug a kid, they say ‘poor woman really likes to have one’. If I stay away from children, they say that I am jealous. I have no idea what to do and how to treat others”. (27 years old, 6 years infertility).

The existence of social supporters against its absence: This category included two subcategories as follow:

1. ***Social supporters:*** The participants stated that the only source of emotional and social supports included friends and relatives, infertile counterparts, religious sessions, visual media and medical staff. The aforementioned sources had a little supportive role in limited cases and conditions. Some participants did not access any source.
  “I go to Quran recitation and ethics gatherings. They really work for me. I will accept that there is a philosophy behind things that have not been granted by God”. (27 years, 6 years infertility).
2. ***The absence of supportive sources:*** The lack of emotional support, information and counseling services, and financial support (in the form of insurance) for treatment needed to bring about an undesirable construction of infertility.
  “No one cares what tensions are imposed by medical staff, people, government, and media on infertile women”. (33 years old, 2 years infertility).
  The participants were deprived of the simplest and the least costly sources of information such as pamphlets and counseling services regarding the treatment process and complications.
  “Whenever I went to the infertility center for an interview, the counselor’s room was either locked or used for treatments. I could never see the center’s psychologist even once”. (Field note).
  “For the government, infertility and cosmetic surgery are alike. The infertility insurance has been introduced at the Iranian parliament several times, but nothing has been done yet. I can ignore a nose job, but infertility is a different story”. (24 years old, 8 years infertility).
  Most participants maintained that media especially TV were able to change people’s attitudes toward treatment methods, but they were inactive.

The characteristics of an infertile woman: This category includes two subcategories as follow:

1. ***Individual biography and infertility characteristics:*** Depending on the variables including age, marriage, duration of infertility, treatment duration, kind of infertility and kind of treatment, the participants’ understandings and experiences form infertility were different.
  “The doctor said that since I was over 30 years old, the likelihood of failure was a little high” (34 years old, 6 years infertility).
  In the first two or three years of marital life, relatives and friends were not that curious about childbearing. However, over time and when infertility continued they started asking questions. The participants who had spent a longer time on treatment faced physical, psychological and financial consequences.
2. ***Economic-social factors:*** Different variables including education, job, family income, place of residence, and the status of the place they lived in resulted in different constructions of infertility. The participants enjoying higher levels of education were more able to use more information resources and applied more problem-solving strategies. However, individuals with lower levels of education applied more passive strategies such as introjection.
  “When someone says something to me, I internalize my depression and sorrow. It has happened that I have cried until sunrise”. (35 years old, 3 years infertility).
  Having a job had a variety of advantages for the participants including an excuse for not having a child, less economic tensions, and searching for relaxation by entertaining oneself with job responsibilities.
  Family income affected following up and duration of the treatment process, selecting the kind of treatment and affording treatment costs. The rural participants’ experiences indicated higher social pressures for childbearing than the urban participants.
  “They know about this stuff very quickly in a village”. (43 years old, 14 years infertility).

Attempts to stabilize life and get rid of being judged by others: The participants applied different strategies in dealing with infertility and its consequences. This category included seven subcategories as follow:

1. ***Conducting treatment measures:*** All participants used different treatment measures.
2. ***Conducting traditional treatments:*** The participants often applied different methods including referral to writers of amulets and prayers, herbal and traditional medicine. Those participants who did not believe in these methods had to follow them since their families strongly believed in these methods.
  “I do not really believe in this stuff. My mother visited a writer of prayers. I carried the prayer just for my mother”. (30 years old, 2 years infertility).
3. ***Spiritual and religious strategies:*** All participants applied different spiritual and religious strategies including praying God, trusting Him and conducting religious vows in different ways for getting pregnant and reducing the complications of infertility.
  “I have waited for such a long time and prayed so much. I say that God will respond to my patients”. (31 years old, 12 years infertility).
  Saying prayers, reading the Quran, different kinds of prayers, Salavat, and religious vows were conducted by the participants.
4. ***Avoidance strategies:*** Some participants avoided pregnant women, newborns, and their infertile counterparts to control or get rid of emotions such as regret, sorrow, and others’ attitudes and behaviors. Moreover, they avoided attending parties and gatherings.
  Some participants adopted strategies such as introjection and wishful thinking. Most participants wished to have twins.
  “I tend to daydream of holding a child’s hand. I go for shopping together. I imagine his face in my dreams that he is such and such”. (27 years old, 6 years infertility).
5. ***Emotional- affective compatibility strategies:*** For adjusting to the infertility consequences, the participants sometimes applied strategies such as crying, sleeping, and talking with their husbands, mothers and relatives.
  “I have a chat with my mother. Her words do impress me. They make me feel calm and relaxed”. (33 years old, 2 years infertility).
  Hanging out with close friends, keeping in touch with infertile counterparts, going shopping, going to work, watching TV, entertaining oneself with housework and listening to music were other methods through which they tried to keep calm.
6. ***Problem-solving strategies:*** These strategies included having an appropriate relationship with one’s husband, proving joys in life, referring to a psychiatrist and ignoring what others said.
  To deal with infertility in a more effective way, some participants searched some information on the issue. They collected information from different sources such as the internet, media, infertility treatment centers, and infertile counterparts. Others attempted to avoid the psychological consequences of infertility through having positive thoughts and conducting social activities.
  “Whatever is God’s will. Husband and wife are more important than children. I try to cope with this problem and be hopeful about the future”. (30 years old, 7 years infertility).
7. ***Hiding infertility from others:*** Some participants hid their infertility from others. They did so to avoid psychological-social pressures imposed by others.
  “I did not want them to know that I use donated eggs”. (31 years old, 6 years Infertility).

Hope for treatment interventions against its damages: This category included two subcategories as follow:

1. ***Hope for treatment interventions:*** Despite all problems they faced during the treatment process, they hoped for successful pregnancies they had either heard about or seen.
  “One of our friends got pregnant when he conducted IVF for the fourth time. I hope that it will work for me this time”. (36 years old, 5 years infertility).
2. ***The psychological consequences of the treatment process:*** The psychological consequences of the treatment process included mental preoccupations, frustration, reduced self-confidence, having difficulty to control one’s behavior in certain conditions, negative thoughts, fear, anxiety, shock, fatigue, disappointment, anger, sorrow and depression.
  “I assumed myself a mother from the moment they implanted the eggs. However, I aborted it”. (35 years old, 3 years infertility).
  “When the treatment process does not go well, I lose my control to the point that I argue over nothing”. (23 years old, 1 year infertility).
  The respondents experienced a shock over hearing the need for treatments such as donated egg or surrogacy.
  Most participants indicated that medical staff does not provide them with enough information about the complete process of treatment. Moreover, medical staff did not care about the psychological consequences of infertility and treatment processes.
  “They would better understand us a little more. They need to know more about their patients, especially those with financial problems and those who are in a bad mood (breaking down and crying) “. (31 years old, 12 years infertility).

The couple’s closer relationship against the threat to life instability: This category had two subcategories as follow:

1. ***The couple’s closer relationship:*** Three participants asserted that infertility and its consequences were the main reasons behind their closer relationships with their husbands.
  “I think that my infertility has made me closer and more intimate to my husband. My husband counts on me and I do too. Therefore, we are now much kinder to each other”. (44 years old, 14 years infertility).
2. ***The likelihood of the disintegration of marital life:*** Most participants stated that if their husbands intended to remarry, they would get divorced. Factors behind the intention of getting divorced included the pressure imposed by relatives on the man to remarry, men’s intention to remarry and infertility despite continuous treatments.
  “Relatives and friends asked him [husband] to divorce me. They used to tell him that they know a widow. He started thinking of remarriage”. (31 years old, 12 years infertility).
  The participants’ experiences indicated that those men who were encouraged to remarry or those who were under pressure to do so were willing to remarry despite having their first wife. However, according to the participants, none of them accepted their husbands’ conditions.

Relieving or adapting to psychological stress against its increase: This category had two subcategories as follow:

1. ***Relieving or adapting to psychological stress:*** According to the participants, adopting certain strategies (previously mentioned in the category of attempts for life stabilization and getting rid of being judged by others) helped adapt themselves with both infertility and its consequences.
2. ***Increased psychological stress:*** Some participants applied silence and introjection strategies against questions raised by others, stigma, and husband’s mental as well as physical violence. While they managed to reduce tensions, they faced increased mental pressures.
  “Many nights I kept quiet for what others said and cried till sunrise. I could not even tell my husband”. (31 years old, 6 years infertility).
  Those participants who adopted social isolation felt lonely in the long term. Those who hid either their infertility or its treatment indicated that they were less judged by others, but they were always anxious for the energy and mental preoccupations they spent on hiding their problems.
  “Now, they doubt what I say. They say: “Child-bearing has nothing to do with husband’s studies. You want to take care of the baby, not your husband”. I am always worried that they might know about everything”. (22 years old, 6 years infertility).

Spiritual growth against its challenges: This category includes two subcategories as follow:

1. ***Spiritual growth:*** Some participants asserted that infertility was a factor for getting closer to God and they were able to learn how to grow closer to God.
  “God makes trouble for those who love more so that they will call Him more to get closer to Him. I am happy about this. Now, I need to communicate better with Him”. (24 years, 8 years infertility).
2. ***Challenging the individual’s spiritual beliefs:*** To some participants, infertility questions the individual’s religious beliefs and spiritual values. God does not respond to their repeated requests made in different ways.
3. “I used to think that God may answer my requests. Now, I think that I am forgotten. He does not love us. I have frequently said prayers. They have turned out to be useless. I just want to ask God how much I should cry. You know, I do not say my prayers as I used to”. (35 years old, 9 years infertility).

## Discussion

The findings of the present study indicated that the participants lived in a society where infertility was associated with social relationships, expectations, and needs. For this reason, most participants indicated that social feedbacks were of great importance to them, also, those participants who enjoyed their husbands and family support were afraid of their future life due to judgments and interventions made by others.

Supportive husbands even knew very little about empathy with the infertile women. Given their wives’ infertility and the treatment process, husbands attempted to provide their wives with peace they needed by hiding their negative feelings and psychological consequences. However, this method was worrying for those women and they felt guilty when found out that their husbands tended to hide their feelings. Those women whose husbands’ performance was a main source of tension adopted strategies such as introjection or silence to reduce or control tensions; their mental pressures increased. African infertile women who adopted ignorance and silence against their husbands’ verbal abuse felt sorrow, depression, and anger (18). In contrast, Australian infertile women used very little introjection and silence (19).

Moreover, the infertile women’s infertility affected individuals including their husbands, family, relatives, friends, medical staff and even policymakers and it is affected by them all as well. Consistent with our study results, the results of a qualitative study reported that almost all interviewees had the support of their own families, especially the mother, but the husband’s family had shown limited support, and many participants faced unfavorable behavior from the husband’s family. These behaviors have affected the quality of life of the women negatively (20), while most Australian infertile women were emotionally supported by families (19). It is possible that one of the reasons for this difference is the high socio-economic level of Australian participants. Studies have also shown that the inappropriate reactions of some friends have caused most infertile women not to participate in various ceremonies such as celebrations, mourning, weddings and birthdays (14, 20). Research findings in Nigeria showed that 64 percent of women suffered from verbal and physical violence by their people around due to infertility (21).

In line with this study, the results of the studies showed that some couples pay a large part of their income for treatment and sometimes have to get the loan or borrow for treatment. While most infertile people are covered by insurance, they cannot use insurance services to treat infertility. Under these conditions, a number of infertile couples cannot be cured due to high costs and financial problems (22, 23). On the other hand, the treatment process disrupted the social function of participants and disrupted their decision making (19). Physicians also do not spend enough time to pay attention to the psychosocial problems of the infertile women (20), and they suffered from complications and poor performance of the treatment team and high costs of treatment (12, 15).

Similar to the results of this study, researchers have shown that even in today’s world, despite many changes in family values, parenting experience is important for women and men, and is a criterion of personal satisfaction, social acceptance and sexual identity (24–26). In some developing countries, where the social security system does not exist, the elderly are completely dependent on their children (27).

Similar to our study, scientific evidence showed that older infertile women with lower education and unemployment have a lower quality of life than younger women with higher education levels (28, 29) and use more traditional treatments (14, 18, 22). English infertile women, like our participants, used different methods to avoid focusing on infertility (2). Participants in some studies stated that they were calm and able to overcome the challenges of life by prayer (2, 30). While Australian infertile women used very few religious strategies, they often used active coping mechanisms including methods to change the source of stress (19). Some of our study participants, like Danish infertile women, used wishful thinking to cope with the consequences of infertility (31).

In line with this study, studies have shown that infertile women tend to avoid exposing stimulating events, such as engaging in fertility-related conversations, relationships with pregnant women and children or engaging with people who make them feel uncomfortable (31, 32), while some infertile women in Southern Vietnam took care of their nephews or neighborhood children (23). In the present study, although the participants reduced their tensions with their relatives, they did not mention caring for the children around them. Given the experiences of the participants, perhaps the reason for this is the fear of being in the spotlight. Like the participants in our study, some of other researchers reported infertile couples tried to avoid social contact with others and had less presence in ceremonies and public places such as mosques so they were not obliged to respond to curiosities about the process and type of treatment (22, 28).

Similar to the beliefs of participants in our study, the results of some studies have shown that infertility may cause positive changes in couples’ relationships and bring them closer to each other (2, 22). But in most studies, fears of losing a shared life, divorce and remarriage of the husband are the most important factors that cause suffering to infertile women, especially in African and Asian countries. Most women considered the husband’s family interference as the main factor in causing these problems (23, 33, 34). In Sweden, half of the participants were separated from their husbands, and in all cases, men had left women (35).

Consistent with our study results, some respondents in qualitative studies said that infertility caused their spiritual relationship to be stronger with God and to feel closer to God (14, 15, 31). Some English infertile women also believed that they were selected by God to experience infertility to grow spiritually and to be strong (2). However, some Christian and Muslim participants felt angry with God for abandoning them, and not responding to their prayers and demands. These conditions were temporary in these women, and after a while, they returned to God and praised for all the things given to them in life (2).

Comparing the experiences of the participants with those of developed countries such as Australia, close friends and colleagues were considered main sources of emotional support for Australian women. Infertile women enjoyed specialist counseling, counterparts’ supportive groups and professional support (19). For example, in 2010, 122 counterparts’ supportive groups were formed from the infertile counterparts in 36 states and regions. Moreover, 39 professional support groups were formed in eight states by the National Infertility Association (36).

The healthcare staff’s performance affected the couple’s marital life in different ways. Some aspects including moral, legal, financial, religious challenges of different treatment methods, the unpredictability of the treatment outcomes and the medical staff’s attitude toward infertility with the medical framework were other environmental aspects influencing the experience of infertility. In this respect, there is a need to providing social supports, empowering infertile women to overcome infertility, and focusing on healthcare support within the psychological-social framework (37, 38).

Data analysis showed infertile women and their husbands had little willingness to other substitute strategies such as adoption, choosing to have no children at all and entertaining oneself with social activities. The participants attempted to overcome infertility and its consequences through depending on their beliefs about infertility, their motivations for childbearing and depending on their abilities, which were developed by the characteristics of infertile women such as biography, infertility characteristics and elements of the economic-social class. No effective sources of support to study such interventions or direct them to be more effective were available. Therefore, the construction of undesirable consequences of infertility in the Iranian society was much stronger than its desirable consequences such as spiritual growth owing to the hardships of infertility and couple’s closer relationships.

A study on the adoption barriers in infertile couples in Iran indicated that 85% of infertile women were against the adoption of a child (39), while in Nigeria, 59.3% refused to adopt a child. Increased agreement of the Nigerians is likely due to increased awareness and knowledge about adoption through the advertisements of the public media (27). In developed societies such as Sweden, infertility is one of the most fundamental issues of everybody’s life and infertile women reduced the consequences of infertility by replacing social activities and taking care of friends and relatives’ children (35).

Social supports and effective coping strategies were two main methods for removing or reducing infertility consequences. Social support is considered a main factor for managing infertility. The participants indicated that infertility policies ignored sources of social supports for infertile women. As long as these sources are ignored, the improvement of the life quality of infertile women and taking any steps toward having a much more desirable interpretation of infertility are impossible.

## Conclusion

The results of the study show that various interactive factors affect the social construction of infertility among infertile women who focus on the central concept of attempts to stabilize life and get rid of being judged by others. One of the ways to achieve this goal is by the help of the social media that is easily accessible to the people in the community. Social media can improve people’s awareness about the psychological-social consequences of infertility, treatments, changing or removing the hidden and obvious taboos in society, and changing the society’s unreasonable expectations from infertile women. It is possible that in this way, infertile people would be less likely to be judged by people around them, and on the other hand, they reduce the undesirable consequences of infertility by learning effective coping strategies through media.
